# Regulation of metabolic products and gene expression in *Fusarium asiaticum* by agmatine addition

**DOI:** 10.1007/s12550-013-0158-y

**Published:** 2013-01-31

**Authors:** Tadahiro Suzuki, Young-Kyung Kim, Hifumi Yoshioka, Yumiko Iwahashi

**Affiliations:** National Food Research Institute, 2-1-12 Kannondai, Tsukuba, Ibaraki 305-8642 Japan

**Keywords:** *Fusarium asiaticum*, DNA microarray, Deoxynivalenol, Acetyl-CoA, 4-aminobutyrate (GABA)

## Abstract

The metabolic products resulting from the cultivation of *F. asiaticum* in agmatine were identified using capillary electrophoresis–time of flight mass spectrometry. Glyoxylic acid was detected from fungal cultures grown in agmatine, while it was absent in control cells. The abundance of other metabolic products of the glycolytic pathway also increased because of agmatine; however, there was no increase in the amounts of pyruvic acid or metabolites from the tricarboxylic acid cycle. Moreover, gene expression levels within *Fusarium asiaticum* exposed to agmatine were analyzed by DNA microarray. Changes in gene expression levels directed the changes in metabolic products. Our results suggest that acetyl coenzyme A, which is a starting substrate for the biosynthesis of deoxynivalenol (DON), was simultaneously produced by activated β-oxidation. Furthermore, the content of 4-aminobutyrate (GABA) was increased in the agmatine addition culture medium. GABA can be synthesized from agmatine through putrescine and might influence the regulation of DON-related genes.

## Introduction

Members of the *Fusarium graminearum* species complex cause fusarium head blight (FHB) in wheat and other small grain cereals, and ear rot in maize (Suga et al. 2008). FHB is one of the most economically important diseases of wheat in the world; it causes yield losses, grain quality reduction, and contamination of grain with trichothecene mycotoxins (Bottalico and Perrone 2002; Goswami and Kistler 2004). The *F. graminearum* species complex consists of at least 13 phylogenetically distinct species (Yli-Mattila et al. 2009). These species tend to produce different, strain-specific trichothecenes including nivalenol, deoxynivalenol (DON), 3-acetyldeoxynivalenol (3ADON), and 15-acetyldeoxynivalnenol (15ADON) (Bottalico and Perrone 2002). Geographically, among members of the *F. graminearum* species complex, 15ADON producers are prevalent in the United States and United Kingdom, whereas 3ADON producers are prevalent in Asia. In particular, the vast majority of *Fusarium asiaticum* are found in temperate regions of Asia, where the annual average temperature is over 15 °C and FHB epidemics occur most frequently (Zhang et al. 2010).

DONs are heat-stable molecules that are not eliminated during the processes currently used in food manufacture. The most efficient way to reduce or prevent DON contamination of food is to limit their biosynthesis by fungi during crop cultivation by using cultivars resistant to *Fusarium* development or mycotoxin accumulation (Champeil et al. 2004). Therefore, understanding the regulation of DON production in pathogens by endogenous and exogenous cues is important for better management of grain storage. At present, no induction and regulation mechanisms for DON production by the *F. graminearum* species complex have been established. Also, the levels of toxin observed in most in vitro culture conditions are several orders of magnitude lower than those measured during infection of live wheat heads and other tissues, suggesting that specific host signals are involved in eliciting toxin production in the pathogen (Boddu et al. 2006; Jiao et al. 2008).

Polyamines are metabolites found in both plants and fungi, and have frequently been associated with stress responses, particularly abiotic responses, in plants. Indeed, altered expression of genes involved in polyamine biosynthesis is often observed during plant defense responses (Alcázar et al. 2006). Host-derived signals mediated by polyamines such as agmatine and putrescine, which are produced during interactions of the host with *F. graminearum* species complex members, can trigger the synthesis of DON (Jiao et al. 2008; Gardiner et al. 2009). In polyamine biosynthesis, agmatine, a cationic compound derived from decarboxylation of the amino acid arginine that serves as the precursor of three major polyamines—putrescine, spermidine, and spermine—has been identified as a strong inducer of DON biosynthesis in *F. graminearum* species complex members (Gardiner et al. 2009). DON biosynthesis is differentially regulated by a large number of fungal genes, including both known and previously uncharacterized putative secondary metabolite biosynthetic gene clusters (Gardiner et al. 2009). However, the role of agmatine in metabolic flow related to the induction of genes encoding enzymes required for DON biosynthesis has not been fully defined.

In this paper, to gain insight into DON synthesis in *F. asiaticum* we examined intracellular activities after addition of DON-inducer agmatine. We also examined the mechanisms regulating intracellular DON.

## Materials and Methods

### Strains, media, growth condition and chemicals

The *F. asiaticum* strain mo285 (isolated from wheat in the field) was used in this study. Although this strain produces DON, it does not produce nivalenol. The strain was maintained on potato dextrose agar purchased from Becton Dickinson (Sparks, MD, USA). Liquid-culture experiments for determining growth rates and DON yields, and performing gene expression microarray and metabolome analysis, were performed in modified Czapek liquid medium (pH 7.7) containing: sucrose 30.0 g/l, NaNO_3_ 2.0 g/l, 15 MgSO_4_·7H_2_O 0.5 g/l, KCl 0.5 g/l, Fe-EDTA 0.01 g/l, K_2_HPO_4_ 1.0 g/l. Agmatine was purchased from Wako Chemical Co. (Osaka, Japan). The conidia or mycelia of *F. asiaticum* strain mo285 were inoculated into potato dextrose liquid medium and preincubated at 25 °C for 3 days at 100 rpm. The conidia or mycelia of the preincubated samples were collected by filtration, and 100 mg of conidia or mycelia were inoculated into medium with or without 2 mM agmatine and incubated at 25 °C for 5 days at 100 rpm.

### Determination of DON yield

Culture conditions were described in the “Strains, media, growth condition and chemicals” section. DON concentration in the culture filtrate was determined using the Veratox DON ELISA kit from Neogen (Lansing, MI, USA) according to the manufacturer’s instructions. Culture filtrates were initially diluted 1:10 in phosphate-buffered saline (PBS; pH 7.5) to provide a detection range of 0.5–6 ppm. Further sequential 1:10 dilutions were made to quantify toxin levels in samples that contained levels above the detection range.

### Total RNA preparation

After collection of the conidia or mycelia by filtration, the samples were frozen and ground in liquid nitrogen using a mortar and pestle. Total RNA was extracted and purified using an RNeasy Plant Mini Kit (Qiagen, Tokyo, Japan). The RNA quality was verified using an Experion automated electrophoresis system (Bio-Rad, Tokyo, Japan). The quantity of RNA in the sample was determined using an Ultrospec 6300 Pro spectrophotometer (GE Healthcare, Tokyo, Japan). Culture conditions were described in the “Strains, media, growth condition and chemicals” section.

### Design and manufacture of the *F. graminearum* genome array

Complete sets of *F. graminearum* sequences and annotations were downloaded from the *Fusarium* comparative sequencing project, Broad Institute of Harvard and MIT (http://www.broadinstitute.org/). We commissioned Agilent Technologies (Tokyo, Japan) to design 60-mer length oligonucleotide probes for a DNA microarray, which consisted of 43,067 probe sets designed from 11,619 genes (chromosome 1: 3,647 genes; chromosome 2: 2,977 genes; chromosome 3: 2,538 genes; chromosome 4: 2,457 genes) of *F. graminearum* (https://earray.chem.agilent.com/earray/). These probes were arranged on an Agilent 4 × 44 K format array slide randomly using e-Array version 5.6 (Agilent Technologies) to create the *F. graminearum* genome array.

### DNA microarray experiments

Total RNAs were analyzed by DNA microarray. Cyanine 3 or cyanine 5 was used to label 1,000 ng of total RNA using the Two-Color Quick Amp Labeling Kit (Agilent Technologies). After confirming the quantities and labeling efficiencies of the cRNA samples, labeled cRNAs were hybridized onto the *F. graminearum* genome array using a Gene Expression Hybridization Kit (Agilent Technologies). Then, the array slides were washed with the Gene Expression Wash Buffer Kit (Agilent Technologies), and scanned with an Agilent Microarray Scanner G2505B (Agilent Technologies). Raw microarray image files were processed using Feature Extraction Software version 9.5.1 (Agilent Technologies). In this process, linear and LOWESS normalizations were carried out to remove the dye biases of the data. Genes classified as induced or reduced were those passing a sample *t*-test (*P* < 0.05). The RNAs extracted from three independent cultures were measured separately.

### Metabolome analysis

Culture conditions were the same as for RNA preparation. Two liters of Milli-Q water including 5 μM internal standard (methionine sulfone and D-camphor-10-sulfonic acid) was added to 1 g of cultivated conidia or mycelia of *F. asiaticum* strain mo285 and agitated. Then, 2 l chloroform and 800 ml Milli-Q water were added and the mixture was agitated before being centrifuged at 2,300 *g* for 5 min at 4 °C. Following centrifugation, the aqueous layer was transferred to a 400-μl filtration tube (Millipore, Ultra-free MC PLHCC HMT centrifugal type filter unit, 5 kDa). The aqueous layer was centrifuged (9,100 *g*, 4 °C, 120 min). Filtrates were dried, and dissolved in 50 μl Milli-Q water. Finally, samples were subjected to mass spectrometry (MS) analysis using an Agilent CE-TOF MS system (Agilent Technologies) in cation and anion modes under the conditions described in Table 1. All experiments were conducted independently three times.

**Table 1:** CE-TOF MS settings used for metabolic product analysis

Cation mode		
	Capillary	Fused silica capillary i.d. 50 μm × 80 cm
	Run buffer	Cation buffer solution (p/n: H3301-1001)
	Rinse buffer	Cation buffer solution (p/n: H3301-1001)
	Sample injection	Pressure injection 50 mbar, 10 s
	CE voltage	Positive, 27 kV
	MS ionization	ESI positive
	MS capillary voltage	4,000 V
	Sheath liquid	HMT sheath liquid (p/n: H3301-1020)
Anion mode		
	Capillary	Fused silica capillary i.d. 50 μm × 80 cm
	Run buffer	Anion buffer solution (p/n: H3301-1001)
	Rinse buffer	Anion buffer solution (p/n: H3301-1001)
	Sample injection	Pressure injection 50 mbar, 10 s
	CE voltage	Positive, 30 kV
	MS ionization	ESI negative
	MS capillary voltage	3,500 V
	Sheath liquid	HMT sheath liquid (p/n: H3301-1020)

Samples were subjected to mass spectrometry (MS) analysis using an Agilent CE-TOF MS system (Agilent Technologies) in the cation and anion modes. Samples were diluted 10-fold for the anion mode or 5-fold for the cation mode

### Data processing and analysis

The peaks detected by CE-TOF MS were automatically extracted using MasterHands automatic integration software ver. 2.9.0.9 (developed at Keio University), with mass electric charge ratio (*m/z*), migration time (MT), and peak area acquired. The acquired peak area value was changed into the relative area value using the following formula:

Collation of *m/z* was performed and all substances were registered into the HMT metabolic product database based on the value of MT. Searches were performed for the detected peaks. The permissible error for searches was set at ±0.5 min (MT) and ±10 ppm (*m/z*).

**Fig. 1:**
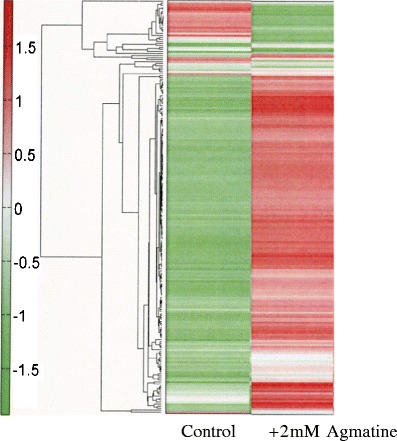
Heat map display. Class clustering was performed using the detected peaks and is presented as a heat map. The horizontal axis shows the sample names and the vertical axis shows the peaks. The distance between peaks is denoted by the tree shape in the figure. Peaks smaller than the average are shown in *deep green*, and those that are larger than the average are *deep red*. The data were standardized by substituting ε (= 0) for N.D. (μ = 0, σ = 1)

We analyzed approximately 108 substances, including amino acids, organic acids, sugar phosphates, and nucleic acids. The concentration of each substance was calculated from an analytical curve based on the peak area (internal standard concentration 200 μM). The metabolic products extracted from three independent cultures were measured separately. The quantitative value expresses the average of each measured value. The heat map is displayed in Fig. 1.

## Results

### Effects of agmatine on DON yield

**Fig. 2:**
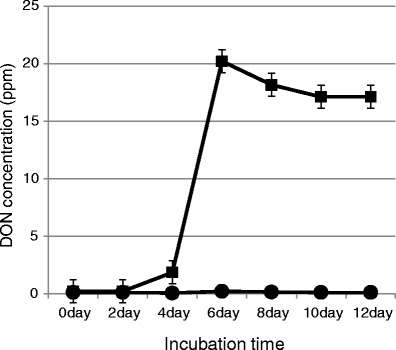
DON yield of *F. asiaticum*. The conidia or mycelia of *F. asiaticum* strain mo285 were inoculated into potato dextrose liquid medium and preincubated at 25 °C for 3 days at 100 rpm. The conidia or mycelia of the preincubated samples were collected by filtration, and 100 mg of conidia or mycelia were inoculated into medium with or without 2 mM agmatine and incubated at 25 °C for 5 days at 100 rpm. Liquid-culture experiments for determining DON yield were performed in modified Czapek liquid medium (pH 7.7); ●: – agmatine (control), ■: + 2 mM agmatine

To perform a biological characterization of the effects of agmatine, we examined DON yields of the *F. asiaticum* strain during incubation. As shown in Fig. 2, the DON yield was noticeably higher in cultures grown in agmatine-containing medium. In the mycelia, which produce DON, many genes related to DON biosynthesis were induced (Table 2). These results were in agreement with the results of a previous study (Gardiner et al. 2009).

**Table 2:** DON-related genes induced by agmatine

Gene ID (mips)^a^	Fold induction	Description
FGSG_00071.2	7.06	Cytochrome P450 mono-oxygenase (TRI1)
FGSG_03534.2	20.8	15-O-acetyltransferase (TRI3)
FGSG_03535.2	12.2	Cytochrome P450 mono-oxygenase (TRI4)
FGSG_03537.2	22.1	Trichodiene synthase (TRI5)
FGSG_03536.2	11.3	Trichodiene biosynthase positive transcription gene (TRI6)
FGSG_03539.2	5.8	Putative trichodiene biosynthase gene (TRI9)
FGSG_03540.2	22.2	Isotrichodermin C-15 hydroxylase (TRI11)
FGSG_03543.2	11.6	Putative trichodiene biosynthase gene (TRI14)

^a^The given gene IDs are entry numbers from the *F. graminearum* genome annotation FG3 by the *Fusarium* comparative sequencing project, Broad Institute of Harvard and MIT (http://www.broadinstitute.org/)

Genes classified as induced or reduced were those passing a sample *t*-test (*P* < 0.05). Each value is the mean of three independent experiments (*n* = 3)

### Glycolytic system

**Fig. 3:**
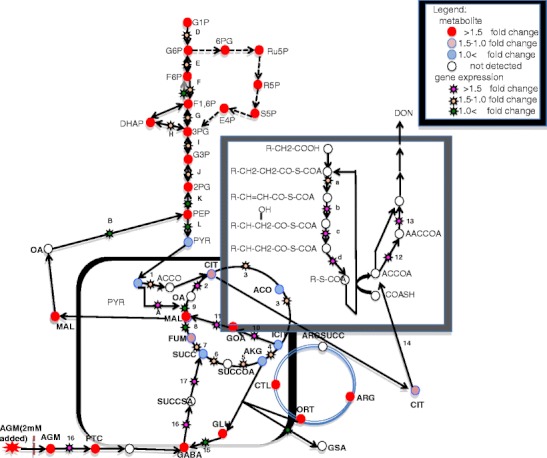
The metabolic pathways of *Fusarium asiaticum* showing changes in metabolite levels and gene expression in response to 2 mM agmatine. *G1P* glucose 1-phosphate, *G6P* glucose-6-phosphate, *F6P* fructose 6-phosphate, *F1,6P* fructose 1,6-bisphosphate, *3PG* glyceraldehyde 3-phosphate, *DHAP* dihydroxyacetone phosphate, *3PG* 3-phosphoglyceric acid, *2PG* 2-phosphoglycerate, *PEP* phosphoenolpyruvate, *PYR* pyruvate, *ACCOA* acetyl-CoA, *CIT* citrate, *ACO* cis-aconitate, *ICIT* isocitrate, *AKG* 2-oxoglutarate, *SUCCOA* succinyl-CoA, *SUC* succinate, *FUM* fumarate, *MAL* malate, *OA* oxaloacetate, *6PG* 6-phosphogluconic acid, *Ru5P* ribulose 5-phosphate, *R5P* ribose 5-phosphate, *S5p* sedoheptulose 7-phosphate, *E4P* erythrose 4-phosphate, *GOA* glyoxylic acid, *PTC* putrescine, *AGM* agmatine, *GABA* 4-aminobutyrate, *Glu* glutamate, *SCCSA* succinate semialdehyde, *ORT* ornithine, *CTL* citrulline, *ARGSUCC* argino-succinate, *ARG* arginate, *GSA* glutamate γ-semialdehyde

Glycolysis-related metabolic products were measured at the time of 2 mM agmatine addition. When agmatine was added to the culture solution, an increase in the levels of metabolic products of the glycolytic system, which begins with phosphorylation of glucose, was observed. Additionally, the amount of each product in the metabolic pathway from glucose 1-phosphoric acid to phosphoenolpyruvic acid increased following exposure to agmatine. Conversely, the level of pyruvic acid was higher in the control cultures than in those in which agmatine was added to the culture medium (Table 3). The levels of the metabolic products in the pentose phosphate pathway were also increased by agmatine (Table 3). Furthermore, we investigated the expression levels of genes relevant to glyconeogenesis and the glycolytic system (Table 4). The expression levels of the genes encoding glycolytic enzymes (Table 4, molecules D–L in Fig. 3) were not significantly changed. However, expression of the gene encoding pyruvate carboxylase, which generates oxaloacetic acid from pyruvic acid in the gluconeogenic pathway, was increased 4.9-fold (Table 4, molecule A in Fig. 3).

**Table 3:** Concentrations of metabolic products in the glycolytic system and the pentose phosphate pathway

	nmol/g	
	Control	2 mM Agmatine
Glycolytic system		
Glucose 1-phosphate	8.1	19.4
Glucose 6-phosphate	110.6	277.2
Fructose 6-phosphate	32.2	72.9
Fructose 1,6-diphosphate	4.5	17.2
Glyceraldehyde 3-phosphate	0	2.1
Dihydroxyacetone phosphate	7.7	15
3-Phosphoglyceric acid	14.5	60.8
2-Phosphoglyceric acid	1.7	6.4
Phosphoenolpyruvic acid	2.4	9.9
Pyruvic acid	162.1	17.5
Pentose phosphate pathway		
6-Phosphogluconic acid	8.1	31.2
Ribulose 5-phosphate	6.7	20.3
Ribose 5-phosphate	2.5	7.3
Sedoheptulose 7-phosphate	13.1	44.7
Erythrose 4-phosphate	6.2	13.2

The concentration of each substance was calculated from an analytical curve based on the peak area. Each metabolic compounds were those passing a sample *t*-test (*P* < 0.05). Values are means of three independent experiments (*n* = 3)

**Table 4:** Expression levels of genes related to glyconeogenesis and glycolysis

	Gene ID (mips)^a^	Fold induction	Description
Glyconeogenesis^b^			
A	FGSG_07075.2	4.9	Pyruvate carboxylase
B	FGSG_08601.2	0.6	Phosphoenolpyruvate carboxykinase
C	FGSG_03127.2	0.6	Fructose-1-6-bisphosphatase
Glycolysis^b^			
D	FGSG_00387.2	1.2	Phosphoglucomutase
E	FGSG_05843.2	1.0	Glucose-6-phosphate isomerase
F	FGSG_09456.2	1.5	6-Phosphofructokinase
G	FGSG_02770.2	0.7	Fructose-bisphosphate aldolase
H	FGSG_06702.2	1.2	Triosephosphate isomerase
I	FGSG_06257.2	1.6	Glyceraldehyde-3-phosphate dehydrogenase
J	FGSG_08922.2	1.1	Phosphoglycerate mutase
K	FGSG_01346.2	0.8	Enolase
L	FGSG_07528.2	0.9	Pyruvate kinase

Each value is the mean of three independent experiments (*n* = 3)

Genes classified as induced or reduced were those passing sample *t*-test (*P* < 0.05)

^a^The given gene IDs are entry numbers from the *F. graminearum* genome annotation FG3 by the *Fusarium* comparative sequencing project, Broad Institute of Harvard and MIT (http://www.broadinstitute.org/)

^b^Letters correspond to those given in Fig. 3

### TCA cycle and the glyoxylate pathway

The expression levels of metabolic products (Table 5) and genes (Table 6) in the TCA cycle were not particularly affected by the addition of agmatine. Glyoxylic acid was detected from the mycelia cultivated in the presence of agmatine (Table 5). Conversely, the level of glyoxylic acid was below the detection limit in the control group (Table 5). The expression levels of the genes encoding isocitrate lyase and malate synthase, which are required for the glyoxylic acid cycle, were increased (Table 6).

**Table 5:** Concentrations of metabolic products of the TCA and glyoxylate cycles

	nmol/g	
	Control	2 mM Agmatine
TCA cycle		
Citrate	1,409.2	1,555.7
cis-Aconitate	41.3	37.5
Isocitrate	59.7	51.9
2-Oxoglutarate	36.2	20.2
Succinate	146.6	126.5
Fumarate	106.9	146
Malate	555.1	872.9
Glyoxylate cycle		
Glyoxylate	0	3.7

The concentration of each substance was calculated from an analytical curve based on the peak area. Each metabolic compounds were those passing a sample *t*-test (*P* < 0.05). Values are means of three independent experiments (*n* = 3)

**Table 6:** Expression of genes related to the TCA cycle, the glyoxylate cycle and acetyl CoA

	Gene ID (mips)^a^	Fold induction	Description
TCA cycle^b^			
1	FGSG_05454.2	1.5	Pyruvate dehydrogenase
2	FGSG_00175.2	1.7	Citrate synthase
3	FGSG_07953.2	1.4	Aconitase family (aconitate hydratase)
4	FGSG_05733.2	1.1	Isocitrate/isopropylmalate dehydrogenase
5	FGSG_04309.2	1.4	2-Oxoglutarate dehydrogenase
6	FGSG_02030.2	1.3	Succinyl-CoA ligase
7	FGSG_05610.2	1.3	Succinate dehydrogenase
8	FGSG_08712.2	0.9	Fumarate hydratase
9	FGSG_02504.2	0.7	Malate dehydrogenase
Glyoxylate cycle^b^			
10	FGSG_09896.2	2.6	Isocitrate lyase
11	FGSG_08700.2	3.3	Malate synthase
Acetyl CoA-related genes^b^			
12	FGSG_09321.2	3.5	Acetyl-CoA acetyltransferase
13	FGSG_09266.2	1.6	Hydroxymethylglutaryl-CoA synthase
14	FGSG_06039.2	2.8	probable ATP citrate lyase subunit 2
β-oxidation^b^			
a	FGSG_02287.2	1.3	Acyl-CoA oxidase
e	FGSG_13398.2	2.5	3-ketoacyl-CoA thiolase B
c	FGSG_03546.2	45.2	3-hydroxyacyl-CoA dehydrogenase
d	FGSG_06457.2	2.0	Enoyl-CoA hydratase/isomerase family

Each value is the mean of three independent experiments (*n* = 3)

Genes classified as induced or reduced were those passing a sample *t*-test (*p* < 0.05)

^a^The given gene IDs are entry numbers from the *F. graminearum* genome annotation FG3 by the *Fusarium* comparative sequencing project, Broad Institute of Harvard and MIT (http://www.broadinstitute.org/)

^b^Numbers correspond to those given in Fig. 3

### Acetyl-CoA-related genes and metabolic products and fatty acid *β*-oxidization

The expression level of the gene encoding isocitric acid dehydrogenase was not affected by the presence of agmatine (Table 6). However, the amount of pyruvic acid in samples decreased less than 11 % after agmatine addition (Table 3), suggesting that pyruvic acid was consumed in some form. Expression of the probable ATP citrate lyase subunit 2 gene, the product of which converts citrate from the matrix of the mitochondria into cytosolic acetyl-CoA, was increased approximately 2.8-fold. The expression of the gene encoding acetyl-CoA carboxylase, which changes acetyl-CoA into malonyl-CoA, was increased approximately 7-fold. Moreover, expression of the gene encoding acetyl-CoA acetyltransferase, which generates acetoacetyl-CoA from acetyl-CoA, was increased 3.5-fold (Table 6). Acetoacetyl-CoA lies at the start of the farnesyl diphosphate biogenetic pathway and is a precursor of DON. The gene cluster encoding the enzymes involved in fatty acid β-oxidization was also strongly induced by agmatine (Table 6).

### GABA-related compounds and genes

Higher levels of γ-amino-n-butyrate (GABA) were found after agmatine addition. Furthermore, agmatinase was induced 29-fold; this enzyme catalyzes the reaction of agmatine to putrescine, which is a precursor of GABA (Table 7). This suggested that the observed GABA was produced via putrescine from the added agmatine.

**Table 7:** Concentration of GABA-related metabolic products and GABA-related gene expression

	nmol/g	
	Control	2 mM Agmatine
Putrescine	20.3	39.8
4-aminobutyrate (GABA)	110.2	191.7
Glutamate	1,616.1	2,930.7
Gene ID (mips)^a^	Fold change	Description
FGSG_01572.2	0.4	Glutamate decarboxylase
FGSG_05446.2	29.4	Agmatinase
FGSG_06751.2	11.2	4-aminobutyrate aminotransferase
FGSG_04196.2	12.3	Succinate-semialdehyde dehydrogenase

The concentration of each substance was calculated from an analytical curve based on the peak area. Values are means of three independent experiments (*n* = 3)

Genes classified as induced or reduced and each metabolic compounds were those passing a sample *t*-test (*P* < 0.05)

^a^The given gene IDs are entry numbers from the *F. graminearum* genome annotation FG3 by the *Fusarium* comparative sequencing project, Broad Institute of Harvard and MIT (http://www.broadinstitute.org/)

## Discussion

Polyamines found in plants have been associated with stress or disease (Walters 2003), and polyamine-related gene expression has been observed during plant defense responses (Haggag and Abd-El-Kareem 2009). It is well known that plants over-express polyamine synthesis genes under infection stress, such as from disease microbes. Examining polyamine synthesis in wheat *in planta*, Gardiner et al. (2010) showed that an arginine decarboxylase gene (which converts arginine to agmatine) was up-regulated during *F. graminearum* infection. Thus, it is very likely that *Fusarium asiaticum* will be exposed to polyamines such as agmatine in vivo. Certain kinds of amine induce production of DON in *Fusarium* spp. (Gardiner et al. 2009) but the mechanisms controlling production of this secondary metabolic product have not been established.

Here, we examined the gene expression profiles and metabolic products of *F. asiaticum* in the presence and absence of agmatine using DNA microarrays and LC-TOF MS. The levels of many of the detected metabolic products were increased by addition of agmatine. Agmatine increased the levels of metabolites generated in the glycolytic system, with the exception of pyruvic acid (Table 3). However, agmatine did not increase the levels of metabolic products of the TCA cycle (Table 5). Interestingly, glyoxylic acid was detected during agmatine-induced DON production in *F. asiaticum*, but not in the absence of agmatine (Table 5). The expression of two genes (isocitrate lyase and malate synthase) associated with the glyoxylate pathway was induced in *F. asiaticum* following agmatine-induced DON production (Table 6). The expression of genes encoding enzymes involved in fatty acid β-oxidation and glyconeogenesis was also induced (Tables 4, and 6).

Furthermore, when agmatine was added, the intracellular GABA content was increased, as was GABA-related gene expression (Table 7). The expression of the glutamate decarboxylase gene, which makes GABA from glutamic acid, was low, and the expression levels of agmatinase (29-fold increase), 4-aminobutyrate aminotransferase (GABAT; 11-fold), and succinate-semialdehyde dehydrogenase (SSADH; 12-fold) were high. This result suggested that the increase in the content of GABA depended on agmatine addition.

Some filamentous fungi are able to utilize GABA as a nutrient source (Kumar and Punekar 1997). Moreover, GABA was reported to be a major nitrogen source during infection of tomato by *Cladosporium fulvum*, in the course of which GABAT and SSADH activities are induced (Solomon and Oliver 2001). On the other hand, in the animal cell, GABA mostly functions as a neurotransmitter. GABA may play a signaling role in the induction of fungal enzymes responsible for the degradation of the plant cell wall (Carapito et al. 2008). In our experiment, added agmatine, simulating the plant resistance response, was taken into the *Fusarium* cell and the concentration of was GABA promoted as expected. This suggested the possibility that GABA could serve as the trigger of a secondary metabolic product such as DON.

Our results suggested that *Fusarium* spp. synthesize DON from acetyl-CoA generated by citrate-pyruvate shuttling and β-oxidation. Acetyl-CoA is generated via three main mechanisms. Firstly, the pyruvate dehydrogenase complex catalyzes the reaction of pyruvate to acetyl-CoA in mitochondoria. In our experiment, although the expression of a citrate dehydrogenase-related gene was increased a little, other metabolites and related enzyme genes of the TCA cycle did not change. This indicated that the conversion of pyruvate to acetyl-CoA had not contributed to production of DON. Secondly, it can be generated by the reaction of acetyl-CoA synthase. Increased expression (5.4-fold; data not shown) of an acetyl-CoA synthase related gene was observed after agmatine addition in our experiment. This indicated that intracellular acetyl-CoA was increased. Lastly, there is the citrate-pyruvate shuttle. For acetyl-CoA to pass through the mitochondrial membrane, acetyl-CoA within the mitochondria is carried to the cytoplasm via this shuttle. Within the mitochondria, oxaloacetate and mitochondrial acetyl-CoA pass through the mitochondrial membrane via tricarboxylate carriers, and are converted to oxaloacetate and acetyl-CoA by ATP citrate lyase (ACL) in the cytoplasm in plants, fungi and animals (Fatland et al. 2002). ATP citrate lyase plays a role in fungal development and production of tricothecenes (Hokyoung et al. 2011). In this study, a probable ATP citrate lyase subunit 2-related gene was increased 2.8-fold. The TCA cycle was in a stationary state while the glycolytic system was promoted. ATP citrate lyase controls these metabolisms and is considered to increase the production of acetyl-CoA. Control of the glycolytic system through acetylation of histones by ACL has also been shown in experiments with mammals and *Gibberella zeae* (Beckner et al. 2010; Hokyoung et al. 2011).

Taking *Aspergillus* spp. as an example, it was reported that polyketide mycotoxins could be produced from acetyl-CoA made using fatty acids within the seeds of the infected host (e.g. corn) in peroxisomes (Maggio-Hall et al. 2005). There are two major metabolic pathways in peroxisomes, the glyoxylate cycle and β-oxidation (Van Roermunda et al. 2003). However, the location of *β*-oxidization and glyoxylate cycle activity was not clear in our experiment (mitochondria or peroxisomes).

We have demonstrated metabolic and transcriptomic changes in *F. asiaticum* in response to agmatine under in vitro conditions, but whether these responses reflect those occurring in vivo will need to be verified in future studies.
